# Combining MSC Exosomes and Cerium Oxide Nanocrystals for Enhanced Dry Eye Syndrome Therapy

**DOI:** 10.3390/pharmaceutics15092301

**Published:** 2023-09-11

**Authors:** Ying Tian, Yiquan Zhang, Jiawei Zhao, Fuxiao Luan, Yingjie Wang, Fan Lai, Defang Ouyang, Yong Tao

**Affiliations:** 1 Department of Ophthalmology, Beijing Chaoyang Hospital, Capital Medical University, Beijing 100020, China; 2 State Key Laboratory for Conservation and Utilization of Bio-Resource in Yunnan, Center for Life Science, School of Life Sciences, Yunnan University, Kunming 650500, China; 3 State Key Laboratory of Quality Research in Chinese Medicine, Institute of Chinese Medical Sciences (ICMS), University of Macau, Macau 999078, China

**Keywords:** dry eye syndrome, mesenchymal stem cell-derived exosomes, reactive oxygen species, cerium oxide nanocrystals, syndrome therapy

## Abstract

Dry eye syndrome (DES) is a prevalent ocular disorder involving diminishe·d tear production and increased tear evaporation, leading to ocular discomfort and potential surface damage. Inflammation and reactive oxygen species (ROS) have been implicated in the pathophysiology of DES. Inflammation is one core cause of the DES vicious cycle. Moreover, there are ROS that regulate inflammation in the cycle from the upstream, which leads to treatment failure in current therapies that merely target inflammation. In this study, we developed a novel therapeutic nanoparticle approach by growing cerium oxide (Ce) nanocrystals in situ on mesenchymal stem cell-derived exosomes (MSCExos), creating MSCExo-Ce. The combined properties of MSCExos and cerium oxide nanocrystals aim to target the “inflammation-ROS-injury” pathological mechanism in DES. We hypothesized that this approach would provide a new treatment option for patients with DES. Our analysis confirmed the successful in situ crystallization of cerium onto MSCExos, and MSCExo-Ce displayed excellent biocompatibility. In vitro and in vivo experiments have demonstrated that MSCExo-Ce promotes corneal cell growth, scavenges ROS, and more effectively suppresses inflammation compared with MSCExos alone. MSCExo-Ce also demonstrated the ability to alleviate DES symptoms and reverse pathological alterations at both the cellular and tissue levels. In conclusion, our findings highlight the potential of MSCExo-Ce as a promising therapeutic candidate for the treatment of DES.

## 1. Introduction

Dry eye syndrome (DES) is a multifaceted ocular disorder characterized by diminished tear production and/or increased tear evaporation, leading to ocular surface dryness and discomfort. Moreover, DES can result in damage to the ocular surface, raising the risk of ocular infections and surface injuries, such as corneal abrasions or ulcers. This can negatively impact vision-related quality of life and interferes with daily activities. The prevalence of DES is high, with up to 34–50% of people suffering from DES [1,2]. According to recent research, an estimated 371 million individuals, comprising approximately 31% of the population that is aged 5 to 89 years in China, are reported to experience symptomatic DES [3]. As a chronic condition that necessitates continuous management, there is an urgent demand for the development of innovative therapies.

One promising approach that has gained significant attention is the use of mesenchymal stem cell-derived exosomes (MSCExos) due to their pro-repair and anti-inflammatory properties. Specifically, MSCExos have demonstrated considerable potential for the treatment of DES, and various mechanisms of action have been explored [4,5]. For example, transforming growth factor β [6], miR-542-3p [7], and miR-21a-5p [8] have been suggested to play essential roles in mediating the therapeutic effects of MSCExos. Despite these advancements, continuing efforts are needed to enhance the effectiveness of MSCExos and expand their functional repertoire for the development of improved therapeutic strategies for DES management.

Meanwhile, the pathological physiology of DES involves multiple processes, encompassing not only inflammation and tissue injury, but also an increase in reactive oxygen species (ROS) levels. These processes play interconnected roles in the development and progression of DES [9]. In brief, during the inflammatory process, a substance known as reactive oxygen species (ROS) is generated, which instigates oxidative stress damage and lipid peroxidation in cell membranes. This sequence of events triggers a transition in cell membrane permeability and induces DNA damage, culminating in widespread damage to the ocular surface and exacerbating the progression of inflammation at various points in the cycle [10,11,12,13,14]. It is important to note that ROS, which include superoxide anion (O_2_•^−^), hydrogen peroxide (H_2_O_2_), and hydroxyl radicals (•OH), are highly reactive oxygen molecules that are produced as byproducts during cellular metabolic reactions [15]. In the normal physiological condition, ROS are crucial for cellular signaling, immune regulation, and apoptosis [16]. However, excessive production of ROS or insufficient antioxidant defense mechanisms can result in oxidative stress, causing cellular damage and dysfunction [17]. Several intervention studies suggest that ROS can be directly targeted in topical therapy for dry eye management [12,18,19]. Recently, novel approaches to neutralize ROS using high-efficiency, nanoscale antioxidants have emerged [20]. Treatments of ROS-related diseases using cerium oxide nanocrystals have been reported, including neurotrauma following stroke [21], sepsis [22], hepatic ischemia-reperfusion injury [23], acute kidney injury [24], and Alzheimer’s disease [25].

In this study, in order to target the “inflammation-ROS-injury” pathological mechanism of DES, we developed a synergistic therapeutic nanoparticle approach for the in situ growth of cerium oxide (Ce) nanocrystals on MSCExos, which we refer to as MSCExo-Ce. While employing MSCExos as a carrier, the MSCExo membrane can function as a template to subtly adjust the nanocrystals’ size and proportion of Ce, thereby amplifying the efficiency of ROS clearance [26]. Concurrently, this confers onto MSCExos the added capacity to mend severely damaged tissues and demonstrate anti-inflammatory activity. Our aim was to probe into the potential of MSCExos and cerium oxide nanocrystals in the treatment of DES and to shed light on the fundamental mechanisms underlying these therapeutic interventions. We hypothesize that this novel therapeutic strategy will offer a new treatment option for patients with DES.

## 2. Materials and Methods

### 2.1. Isolation and Characterization of MSCExos

Mesenchymal stem cells (MSCs) were segregated and refined from nucleated cells derived from murine bone marrow. Following a period of 72 h, cells that did not adhere were discarded, while the adherent cells continued to be cultivated for an additional span of 16 days in α-MEM medium (Gibco, Waltham, MA, USA). All cell cultures were sustained in a medium that incorporated 10% exosome-depleted FBS (Gibco), 1% (*v*/*v*) penicillin, and 1% (*v*/*v*) streptomycin. The cell cultures were incubated at 37 °C in a 5% CO_2_ atmosphere. Exosomes were extracted from MSC-conditioned media in accordance with a previously established protocol. Briefly, the cell culture supernatant underwent centrifugation at 300× *g* for 10 min at 4 °C to remove cells, 2000× *g* for 10 min at 4 °C to eliminate dead cells, 10,000× *g* for 30 min at 4 °C to clear cell debris, and two rounds of 100,000× *g* for 70 min at 4 °C to isolate exosomes [27].

### 2.2. MSCExo-Ce Preparation

Ce(NO_3_)_3_·6H_2_O was incorporated into MSCExo (containing 1012 vesicles/mL) to reach a final concentration of 1 mM. Subsequently, the amalgamated solution was incubated at 37 °C for an hour within a temperature-regulated container, which promoted the absorption of Ce^3+^ onto the MSCExo surface and resulted in MSCExo-Ce^3+^. Following this step, the MSCExo-Ce^3+^ was purified by filtering it through a 100 kDa centrifugal filter device (Amicon Ultra-0.5, Millipore Co., Darmstadt, Germany) three times at 4500× *g* for 15 min at 4 °C to eliminate any unbound Ce^3+^. The purified MSCExo-Ce^3+^ was then resuspended in 25 mM of 2-[4-(2-hydroxyethyl) piperazin-1-yl] ethanesulfonic acid (HEPES) buffer and incubated overnight at 4 °C. To encourage the formation of smaller CeO_2_ particles and evade potential damage to membrane proteins, the pH was adjusted to 7.4 for CeO_2_ growth. Following overnight incubation, Ce^3+^ crystallized on the MSCExo surface, resulting in the formation of MSCExo-Ce. The MSCExo-Ce was further purified by filtering it three times using a 100 kDa centrifugal filter device at 4500× *g* for 15 min at 4 °C.

### 2.3. MSCExo-Ce Characterization

High-resolution transmission electron microscopy (HR-TEM) and energy dispersive spectroscopy (EDS) analyses were performed using a JEM-2100F microscope set at an accelerating voltage of 200 kV. The particle size distribution and zeta potential were evaluated through nanoparticle tracking analysis (NTA) using the ZetaView instrument (Particle Metrix, Inning am Ammersee, Germany). The quantity of cerium that was incorporated into MSCExo-Ce was established via inductively coupled plasma mass spectrometry (ICP-MS) using an iCAP Qc system (Thermo Fisher Scientific, Waltham, MA, USA), and the cerium was subsequently normalized based on the vesicle count.

### 2.4. ROS-Scavenging Activity Assay

The scavenging activities of hydroxyl radicals and superoxide anions were evaluated using a microscale hydroxyl free radical scavenging capacity assay kit and a microscale superoxide anion assay kit while following the manufacturer’s instructions. MSCExo and various concentrations of MSCExo-Ce were incorporated into each assay, which was then examined by measuring the fluorescence intensities. The nitrogen free radical scavenging activity was evaluated using 1,1-diphenyl-2-picrylhydrazyl (DPPH). DPPH was prepared at a concentration of 10 M, and MSCExo along with distinct concentrations of MSCExo-Ce were introduced. The resulting mixture was allowed to incubate at room temperature for a duration of 30 min, and absorbance readings were subsequently obtained at 526 nm.

### 2.5. Uptake of MSCExo-Ce by Human Corneal Epithelial Cells (HCECs)

The MSCExo-Ce solution underwent Cy5 staining for 60 min at 37 °C. Unbound dye was subsequently removed utilizing Exosome Spin Columns (Thermo Fisher). HCECs were exposed to MSCExo-Ce for 12 h, after which they were fixed and imaged.

### 2.6. In Vitro Scratch Wound Assay

HCECs were seeded into 24-well plates at a density of 2.5 × 10^5^ cells per well and were cultured overnight. Subsequently, a sterile 10 μL pipette tip was utilized to create a scratch on the cell monolayer, which was then washed twice using phosphate buffer saline (PBS) to eliminate any detached cells. Following this step, the cells were treated using various drugs, including PBS, MSCExo (100 μg/mL), and MSCExo-Ce (100 μg/mL), in fresh medium, and the cells were co-incubated for a period of 12 h. The cell cytoskeleton was stained using FITC-conjugated phalloidin (Beijing Solarbio Science & Technology, Beijing, China), and confocal laser scanning microscopy (CLSM) (Leica, Wetzlar, Germany) was employed to capture images of the scratched area.

### 2.7. In Vitro Mitochondrial ROS Scavenging

HCECs were seeded into 12-well plates at a density of 5 × 10^5^ cells per well and allowed to culture for 12 h. Subsequently, cells were treated with PBS, MSCExo (100 μg/mL), and MSCExo-Ce (100 μg/mL), and incubation was continued for an additional 12 h. Following a 10 min treatment with H_2_O_2_ (400 μM), mitochondrial ROS were generated. Subsequently, the 2′-7′dichlorofluorescein diacetate (DCFH-DA) probe was incorporated and allowed to incubate for 30 min at 37 °C in a light-protected environment for the detection of mitochondrial ROS. For comparison, one group that did not receive any treatment was established as the negative control. Fluorescence microscopy images were captured by employing a CLSM analysis (Leica, Germany).

### 2.8. Cytotoxicity Study

HCECs were plated in 96-well plates at a density of 10^4^ and incubated for 12 h. Then the medium was placed with 100 μL of DMEM basic (1X) (Gibco) containing different concentrations of MSCExo-Ce (0, 50, 100, 200, μg mL^−1^). After culturing for 24 h, the cell viability was measured using Cell Counting Kit-8 (CCK-8) (Solarbio Life Sciences, Beijing, China) and a live/dead assay (Beyotime Biotechnology, Yancheng, China).

### 2.9. Dry Eye Model Mice

C57BL/6 male mice (6–8 weeks old, weighing 20 ± 2 g) were sourced from Beijing Vital River Laboratory Animal Technology Co., Ltd., for the creation of dry eye disease (DES) models. The mice were housed in an environment with controlled conditions, wherein we maintained a steady temperature of 22 ± 1 °C and a uniform light/dark cycle (12 h/12 h) with unrestricted access to food and water. Benzalkonium chloride (BAC) (Sigma-Aldrich, St Louis, MO, USA) was employed to induce DES in the experimental mice. In brief, the right eyes of the mice received 5 μL of 0.2% BAC eye drops (*w*/*v*) administered twice daily for a duration of 7 days. Subsequently, the mice were randomly allocated into three groups and treated with a topical application of either 10 μL of PBS, MSC-Exo, or MSCExo-Ce twice daily. Additionally, the healthy eye that did not undergo any eyedrop treatment served as the control group.

### 2.10. Cotton Thread Test

Pentobarbital (0.05 mg per g body weight) was administered via intraperitoneal injection. After a 5 min waiting period, the phenol cotton thread test (Zone-Quick, Showa Yakuhin Kako, Tokyo, Japan) was conducted to assess tear production. Using a tweezer, the thread was carefully placed at the lateral canthus for 30 s. As tears were absorbed by the cotton, it turned red, and the wetted length was promptly measured.

### 2.11. Fluorescein Staining

Fluorescein staining was conducted using 5 µL of 0.5% sodium fluorescein solution, which was introduced into the conjunctival sac. The extent of the damage was assessed using a cobalt blue filter and separated into four quadrants, each receiving a grade ranging from 0 to 4. In brief, the following grading system was applied to each quadrant: (0) absence of staining; (1) mild punctate staining with fewer than 30 spots; (2) punctate staining with more than 30 spots, but not widespread; (3) extensive diffuse staining without a distinct plaque; and (4) presence of a positive fluorescein plaque-staining score. To obtain the final fluorescein score, the total scores of all quadrants were combined. Three right eyes from each group underwent examination, and the mean value was employed for data analysis.

### 2.12. ELISA of Tear Samples

Tear fluid specimens were collected by irrigating the ocular surface with PBS, which was followed by microcapillary tube collection. In detail, a 10 μL droplet of PBS intended for mice was administered to the eye surface for a duration of 10 s with subsequent capillary retrieval. Healthy eye tear fluid samples served as the control group. These tear fluid samples were promptly preserved at −80 °C until further analysis. The concentrations of cytokine IL-1β within the samples were determined using an ELISA kit (Beijing Solarbio Science & Technology).

### 2.13. Hematoxylin and Eosin Staining

Following euthanization, the eyes from different groups were collected and fixed using an FAS eye fixation solution (Wuhan Servicebio Technology Co., Ltd., Wuhan, China). The eyes were then embedded in paraffin and sectioned into 3 µm-thick vertical slices. These sections were rinsed in deionized water for 5 min and stained using hematoxylin and eosin as per standard protocol. The morphologies of the corneas were examined using an EVOS FLc microscope.

### 2.14. In Vivo ROS Scavenging and TUNEL Assay

The eyeballs were meticulously extracted from their orbits and immediately frozen in dry ice at optimal cutting temperature. Fresh 10 μm frozen sections were prepared. DHE (Dihydroethidium) served as the staining probe for ROS in the cornea. For this purpose, cornea flat mounts were incubated with DHE at 37 °C for a duration of 30 min. TUNEL staining was performed using the in situ Cell Death Detection Kit Fluorescein (Roche, IN, USA) while following the manufacturer’s guidelines. Subsequently, the sections were imaged using a CLSM analysis (Leica, Germany).

### 2.15. Distribution of MSCExo and MSCExo-Ce In Vivo

The MSCExo and MSCExo-Ce were labeled using Cy5 for 60 min at 37 °C, respectively; they were dropped on the ocular surface of mice twice a day, and we extracted the eyeballs from their orbits and immediately froze them in dry ice at optimal cutting temperature while preparing fresh 10 μm frozen sections. Subsequently, the sections were imaged using a CLSM analysis (Leica, Germany).

### 2.16. Safety Evaluation

We randomly assigned 20 mice into 2 groups, with 10 mice serving as the healthy control group and the remaining 10 mice being treated with a topical instillation of 10 μL of MSCExo-Ce on their right eye twice daily for a duration of 7 consecutive days. On the 7th day, the right eyes of the mice were evaluated using a tonometer. Furthermore, on day 30, significant organs, such as the liver, lungs, heart, kidneys, and spleen, were rapidly harvested from the mice after euthanasia. These organs were then processed into H&E-stained specimens to further assess in vivo biosafety.

### 2.17. Statistical Analysis

The data were presented as the means ± SD. The data presentations were carried out using GraphPad Prism 9.0 software (La Jolla, CA, USA). Comparisons involving only two groups were conducted using a two-tailed unpaired Student’s *t*-test, while those involving more than two groups were carried out using a one-way analysis of variance (ANOVA) test. *p*-values < 0.05 were deemed significant, and * *p* < 0.05, ** *p* < 0.01, *** *p* < 0.001, and **** *p* < 0.0001 were considered statistically significant.

## 3. Results

### 3.1. Preparation and Characterization of MSCExo-Ce

**Figure 1:**
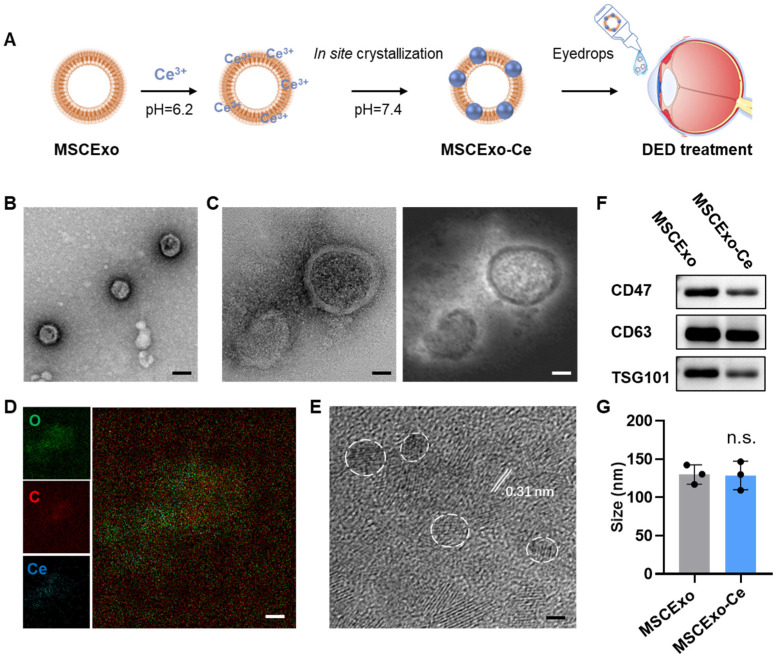
Preparation and characterizations of in situ cerium oxide crystallized mesenchymal stem cell exosomes (MSCExo-Ce). (**A**) Schematic for the fabrication of MSCExo-Ce. Exosomes were incubated in saline at 37 °C with 1 mM of Ce^3+^ for 1 h and then ultra-filtrated to remove excess Ce^3+^. Cerium oxide crystals were crystalized on the Exo membrane upon pH adjustment to 7.4. The fabricated MSCExo-Ce was preserved as the eyedrops formulation for DES treatment. (**B**) TEM imaging of MSCExo-Ce stained with uranyl acetate. The scale bar is 100 nm. (**C**) High-resolution TEM (HR-TEM) imaging of MSCExo-Ce (**left**) and the corresponding dark field imaging (**right**). The scale bar is 20 nm. (**D**) Elemental mapping imaging of MSCExo-Ce. The scale bar is 20 nm. (**E**) HR-TEM imaging of MSCExo-Ce, with the lattice spacing being indicated by the white line. White circles indicate cerium oxide nanocrystals. The scale bar is 2 nm. (**F**) Western blotting analysis of MSCExo-Ce. CD47, CD63, and TSG101 are classical markers for exosomes. (**G**) Size distribution of MSC-Exo and MSCExo-Ce (*n* = 3 biologically independent samples). The data in G are presented as the means ± SD and are assessed by a two-tailed unpaired Student’s *t*-test (n.s.: not significant).

We employed a one-pot method for the in situ synthesis of cerium nanocrystals on MSCExo, as detailed in our previous work [28]. In brief, cell supernatant from the MSC culturing was collected, and exosomes were purified via ultracentrifugation. The MSCExos were then incubated in a Ce^3+^ solution (1 mM, pH = 6.2) to facilitate absorption onto the MSCExo membranes through electrostatic interactions. After removing excess Ce^3+^, the purified MSCExo-Ce^3+^ was then resuspended in a 25 mM HEPES buffer and incubated overnight at 4 °C (pH = 7.4); cerium oxide nanocrystals were grown in situ on the MSC membranes. The developed MSCExo-Ce was mixed with saline in an eyedrop bottle (Figure 1A). Transmission electron microscopy (TEM) and high-resolution TEM (HR-TEM) were used to capture the morphology of MSCExo-Ce, which exhibited a similar cup-like shape to naïve exosomes (Figure 1B). Dark field imaging revealed metal crystals surrounding the exosomes (Figure 1C), which were confirmed as cerium through elemental mapping imaging (Figure 1D). The diameter of cerium nanocrystals was measured to be less than 3 nm, with a lattice spacing of 0.31 nm (Figure 1E). Taken together, MSCExo-Ce eyedrops were successfully produced and thoroughly characterized.

### 3.2. ROS Scavenging Capacities and Storage Stability of MSCExo-Ce

**Figure 2:**
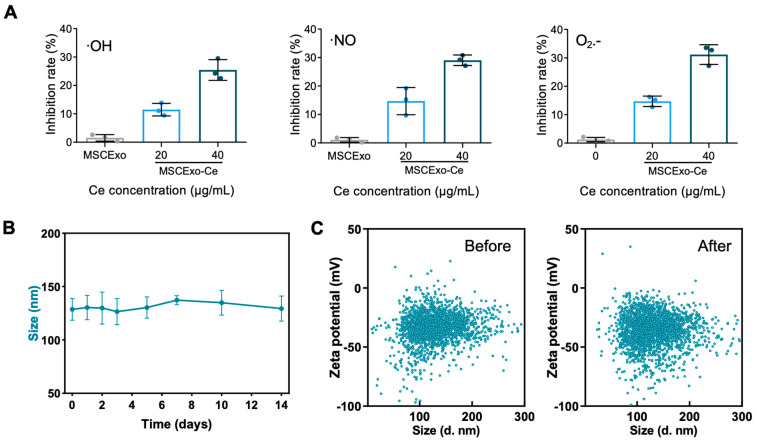
ROS scavenging ability and storage stability of MSCExo-Ce. (**A**) Quantitative analysis of the ability of MSCExo and MSCExo-Ce to scavenge •OH, •NO, and O_2_•^−^ in a dose-dependent manner (*n* = 3 biologically independent samples). (**B**) Particle size of MSCExo-Ce stored for 14 days in PBS at 4 °C (*n* = 3 biologically independent samples). (**C**) Particle size and corresponding zeta potential of MSCExo-Ce before (**left**) and after (**right**) lyophilization/rehydration (Lyo/Reh). Data in (**A**,**B**) are presented as the means ± SD.

After obtained the MSCExo-Ce, we first investigated their ROS scavenging capabilities. Commercial detection kits were employed to measure the ROS scavenging ability. As predicted, MSCExo-Ce demonstrated superior scavenging capacity for hydroxyl radicals, nitrogen free radicals, and superoxide anions compared with naïve exosomes, which is due to the ultrasmall nanocrystals on the MSC membranes (Figure 2A). Furthermore, a 14-day observation of MSCExo-Ce size distribution in PBS was conducted to confirm its stability. No notable size changes were observed during storage in saline at 4 °C (Figure 2B). Similarly, no significant shifts in the particle size or zeta potential were detected before or after lyophilization/rehydration cycles (Figure 2C). These findings indicate that MSCExo-Ce possesses excellent ROS scavenging ability and storage stability.

### 3.3. Effect of MSCExo-Ce on Corneal Repair and ROS Scavenging In Vitro

**Figure 3:**
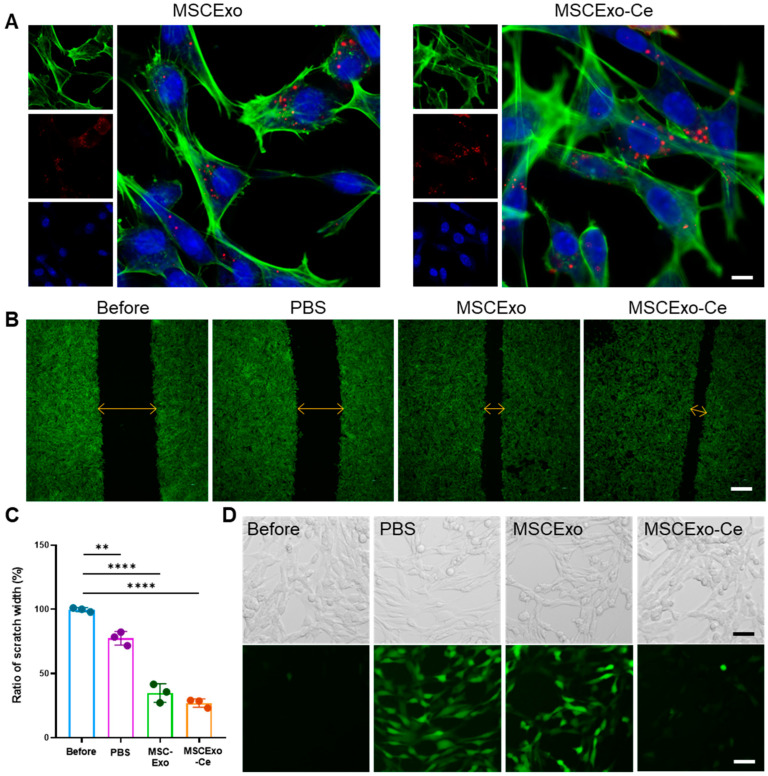
Re-epithelialization properties and ROS scavenging ability of MSCExo-Ce in vitro. (**A**) Confocal laser scanning microscope (CLSM) images of intracellular internalization of MSCExo or MSCExo-Ce in HCECs (Green: phalloidin-labelled cell cytoskeleton, red: Cy5-labelled MSCExo or MSCExo-Ce, blue: DAPI-labelled cell nucleus). The scale bar is 10 μm. (**B**) CLSM images of the scratch assay of HCECs treated with PBS, MSCExo, and MSCExo-Ce. Green: phalloidin-labelled cell cytoskeleton. The scale bar is 100 μm. (**C**) Quantitative analysis of scratch width in (**B**). Data in (**C**) are presented as the means ± SD and are assessed via a one-way ANOVA test. **** *p* < 0.0001; ** *p* < 0.01; n.s. means not significant. (**D**) Representative CLSM images of ROS scavenging in HCECs treated with PBS, MSCExo, and MSCExo-Ce. The upper and lower panels show bright field and CLSM images, respectively. Green: 2′,7′-dichlorofluorescein (DCF) probe. The scale bar is 50 μm.

Before examining the corneal repair and ROS removal capabilities of MSCExo-Ce, we assessed their uptake by HCECs. As illustrated in Figure 3A, MSCExo or MSCExo-Ce that were labeled with Cy5 and exhibited red fluorescence were primarily localized around the perinuclear region, as indicated by the blue fluorescence. The cerium crystallization on the surface of MSCExo did not influence MSCExo-Ce’s cellular internalization by HCECs. To investigate the in vitro corneal repair effect of MSCExo-Ce, a scratch assay on HCEC monolayers was performed while using equivalent amounts of MSCExo and PBS as controls. We found that re-epithelialization treated with MSCExo-Ce was significantly accelerated in monolayers, with a remaining wound area of 27.63 ± 2.031% as compared with PBS (77.53 ± 5.131%) or MSCExo (34.83 ± 5.702%) treatments (Figure 3C). These results highlight the exceptional re-epithelialization properties of MSCExo-Ce, which is attributable to the well-established migration-promoting capabilities of MSCExo.

Besides corneal repair, ROS scavenging is vital due to the anterior segment of the eye’s susceptibility to oxidative stress, which produces excessive unstable ROS that inflict cellular damage in DES. In this study, we scrutinized the intracellular ROS scavenging efficacy of MSCExo-Ce in H_2_O_2_-treated HCECs. As depicted in Figure 3D, both the PBS and MSCExo groups exhibited pronounced green fluorescence of DCF (2′,7′-dichlorofluorescein), signifying higher ROS content in the HCECs. In contrast, the green fluorescence intensity was minimal with MSCExo-Ce treatment, highlighting its superior intracellular ROS scavenging capability. Furthermore, MSCExo-Ce showed excellent biocompatibility on HCECs, even at the different concentrations of 0, 50, 100, 200 μg mL^−1^, as demonstrated by the CCK-8 assay (Figure S1A,B). These results suggest that MSCExo-Ce possesses both pro-wound healing and ROS scavenging abilities.

### 3.4. Evaluating the Therapeutic Potential of MSCExo-Ce for DES Using BAC-Induced Mice

**Figure 4:**
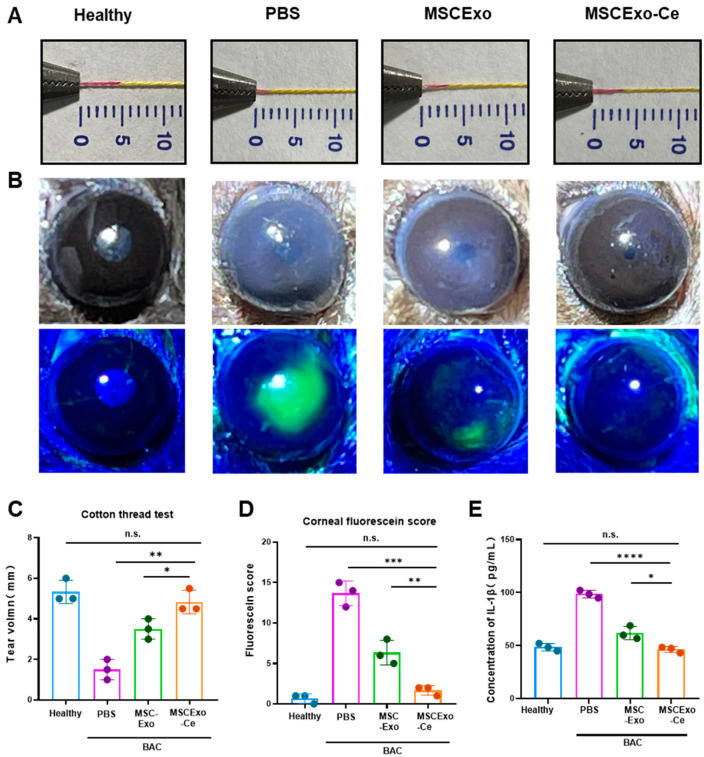
MSCExo-Ce suppresses DES in BAC-induced mice. (**A**) Cotton thread measurement of the wetting length. The normal mice are the healthy group, the BAC-induced mice treated with PBS are the PBS group, the BAC-induced mice treated with MSCExo (100 μg/mL) are the MSCExo group, and the BAC-induced mice treated with MSCExo-Ce (100 μg/mL) are the MSCExo-Ce group. (**B**) Slit lamp and fluorescein staining showing the representative ocular changes of BAC-induced mice in each group after treatment with PBS, MSC-Exo, and MSCExo-Ce. (**C**) Quantitative analysis of the wetting length in (**A**). (**D**) Mean staining score of the eye surface in (**B**). (**E**) ELISA measurement of inflammatory cytokines IL-1β in murine tears. The data in (**C**–**E**) are presented as the means ± SD and are assessed via a one-way ANOVA test. **** *p* < 0.0001; *** *p* < 0.001; ** *p* < 0.01; * *p* < 0.05; n.s. means not significant.

To investigate the effectiveness of MSCExo-Ce as a potential treatment for DES, a BAC-induced dry eye mouse model was employed. This model exhibits the key clinical characteristics of DES and is well established for assessing treatment responses [29,30,31]. C57BL/6J mice received 5 µL eye drops of 0.2% BAC twice daily for 7 days to induce the BAC mouse model. The mice were then randomly assigned to different treatment groups, receiving 5 µL of eye drops per eye twice daily for 7 days. After the treatment, tear secretion measurements using the cotton thread test revealed a significant reduction in tear production in BAC-induced mice treated with phosphate-buffered saline (PBS) compared with the healthy mice without DES. In contrast, both MSC-Exo- and MSCExo-Ce-treated groups displayed increased tear secretion, especially for MSCExo-Ce-treated mice, which showed tear secretion levels that were similar to the healthy group (Figure 4A,C). This finding suggests that MSCExo-Ce may be effective at alleviating DES symptoms in BAC-induced mice. Corneal surface damage evaluation using fluorescein staining (Figure 4B,D) demonstrated that BAC-induced mice treated with PBS or MSC-Exo exhibited corneal edema, reduced transparency, and epithelial defects, while MSCExo-Ce-treated mice displayed a transparent cornea with less fluorescein staining. An inflammatory mediator analysis of mouse tears (Figure 4E) showed significantly reduced IL-1β concentration in the MSC-Exo- and MSCExo-Ce-treated groups compared with the PBS treated group, indicating reduced inflammation.

### 3.5. Histological Evidence of MSCExo-Ce’s Therapeutic Impact on DES in BAC-Induced Mice

**Figure 5:**
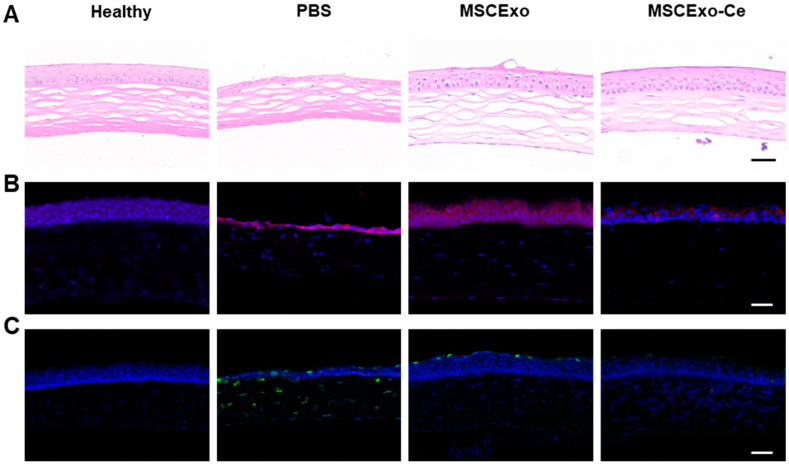
Histological manifestations of MSCExo-Ce in treating DES in BAC-induced mice. (**A**) Representative H&E staining images of the cornea after the different treatments with PBS, MSCExo, and MSCExo-Ce. The scale bar is 100 μm. (**B**) Evaluations of oxidative stress (ROS) by immunostaining on the corneal epithelial cells in the mice eyes after diverse therapies for DES. Red: dihydroethidium as the probe of ROS. The scale bar is 100 μm. (**C**) Apoptosis by TUNEL immunostaining on the corneal epithelial cells in the mice eyes after diverse therapies for DES. Green: FITC–dUTP. The scale bar is 100 μm.

At the onset of DES, the ocular surface experiences extensive inflammatory damage, which leads to epithelial defects, stromal swelling, and decreased tear volume. A histological analysis revealed that the MSCExo-Ce treatment effectively preserved corneal epithelium integrity, increased central cornea and epithelial layer thickness, and restored normal corneal structure compared with PBS- or MSC-Exo-treated mice (Figure 5A). Elevated levels of reactive oxygen species (ROS) in DES contribute to ocular surface inflammation and tear film hyperosmolarity, which results in corneal damage, particularly to the epithelial layer. As demonstrated in Figure 5B, BAC-induced DES modeling led to a significant increase in ROS intensity on the corneal surface and in the corneal epithelia. However, both the healthy and MSCExo-Ce-treated groups exhibited minimal fluorescence, indicating effective ROS scavenging by MSCExo-Ce, even if the MSCExo group still has a considerable amount of fluorescent ROS indicators due to the insufficient ability of MSCExo to scavenge ROS on its own. As shown in Figure S2, there were no significant differences in the depth of penetration, ocular retention, and resistance to degradation. Additionally, an immunofluorescence analysis showed that MSCExo-Ce inhibited TUNEL^+^ cellular apoptosis (Figure 5C), suggesting that MSCExo-Ce promotes ocular surface repair during dry eye by scavenging excessive ROS and preserving corneal structures.

### 3.6. Safety Assessment of MSCExo-Ce

**Figure 6:**
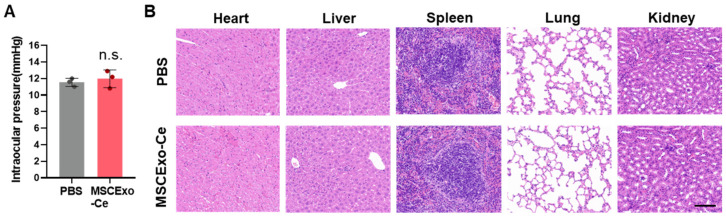
Safety profiles of MSCExo-Ce. (**A**) Measurement of the intraocular pressure using Goldmann applanation tonometry for the assessment of local responses to MSCExo-Ce. (*n* = 3 eyes). (**B**) Major organ (heart, liver, spleen, lung, and kidney) sections stained with hematoxylin and eosin (H&E) were analyzed for the systemic toxicity evaluation. The scale bar is 100 μm. Data in (**A**) are presented as the means ± SD and were assessed using a two-tailed unpaired Student’s *t*-test (n.s.: not significant).

Considering the promising therapeutic outcomes, it is essential to evaluate the biosafety of MSCExo-Ce for potential clinical trials. An in vivo safety evaluation (Figure 6A) showed that mice treated with PBS or MSCExo-Ce maintained stable intraocular pressure on day 7 post-treatment. Moreover, after administering the treatment twice daily for 30 consecutive days, gross necropsies and histological analyses of the major organs were conducted to assess the long-term toxicity of MSCExo-Ce treatment. As depicted in Figure 6B, no gross or histopathological abnormalities or lesions were found in the heart, liver, kidney, spleen, or lungs. These results indicate that treatment with MSCExo-Ce possesses high biocompatibility and excellent ocular tolerance, suggesting its potential as a safe therapeutic approach for future clinical applications.

## 4. Discussion

A growing body of research indicates that native MSCExos possess the ability to enhance wound healing and modulate immune responses. Consequently, numerous researchers have been investigating the potential therapeutic application of MSCExos for the repair and anti-inflammatory treatment of DES [32,33]. Recent evidence suggests that ROS plays a crucial role in the pathogenesis of DES, emphasizing potential therapeutic targets that are aimed at reducing oxidative stress and restoring redox homeostasis in ocular tissues [34,35]. Oxidative stress induced by ROS can directly damage lipid membranes and proteins in the tear film and ocular tissues [13]. Lipid peroxidation, which is initiated by ROS, can alter the tear film composition and compromise its stability, thus contributing to tear film instability and increased evaporation. Furthermore, ROS can induce inflammation in the ocular surface epithelium, worsening DES cases [36]. ROS can activate pro-inflammatory signaling pathways, leading to the release of inflammatory mediators and cytokines [37]. The resulting chronic low-grade inflammation further damages the ocular surface, impairs tear film integrity, and exacerbates tear evaporation. Heightened ROS production leads to oxidative stress and inflammation in ocular surface tissues; inflammation, in turn, further boosts ROS production, perpetuating a vicious cycle. Therefore, targeting ROS not only provides direct relief of DES symptoms but also disrupts the “ROS-inflammation” vicious cycle, leading to a comprehensive alleviation of DES.

Due to the pivotal role of ROS in inflammation progression and oxidative stress-mediated damage, ROS elimination through potent antioxidants has emerged as a highly promising therapeutic strategy for effectively managing inflammatory disorders [38,39,40]. Over the past decade, nanotechnology has been extensively employed in inflammation research, particularly in the development of “nano-antioxidants” [41,42]. These nano-antioxidants hold immense promise as a potential therapeutic approach for effectively scavenging the ROS generated during inflammation, thereby protecting tissues from oxidative damage. In this study, we presented a novel solution to overcome existing challenges that are hindering the application of nano-antioxidants by using cerium as a testbed compound. In contrast to the traditional hydrothermal method used for synthesizing cerium oxide nanocrystals [43,44], our innovative one-pot approach, which utilizes exosomes as templates, offers significant advantages, including simplicity, mild reaction conditions, and prevention of damage to exosome components. Importantly, the in situ crystallization process yielded extremely small cerium oxide nanocrystals, with diameters spanning almost below 5 nm (Figure 1E), resulting in a highly efficient ROS scavenging performance, as it has been reported that the size of cerium oxide nanocrystals is a critical factor for ROS scavenging efficiency [45,46].

In this study, we successfully developed a novel eyedrop formulation, herein named MSCExo-Ce, by synergistically combining the regenerative and anti-inflammatory properties of MSCExo with the ROS-scavenging capability of cerium. Our primary objective was to assess the potential therapeutic efficacy of MSCExo-Ce in treating DES. The characterization analysis confirmed the successful in situ crystallization of cerium onto MSCExo. Moreover, MSCExo-Ce demonstrated excellent biocompatibility, even at high concentrations of up to 100 μg/mL of cerium. Subsequent in vitro and in vivo experiments revealed that MSCExo-Ce displayed remarkable abilities to promote corneal cell growth, scavenge ROS, and suppress inflammation, surpassing the abilities of MSCExo alone. These findings provide strong evidence for the potential of MSCExo-Ce as an effective therapeutic intervention for DES. Notably, MSCExo-Ce exhibited the ability to alleviate DES symptoms and effectively reverse the pathological alterations associated with DES, both at the cellular and tissue levels. In conclusion, our findings reinforce the potential use of MSCExo-Ce as a promising therapeutic candidate in a new class of drugs for the treatment of DES. However, due to the complex nature of DES, further investigations are needed to elucidate the precise mechanism by which MSCExo-Ce exerts its therapeutic effects in DES.

## Data Availability

The datasets generated and/or analyzed during the current study are not publicly available due to the university rules and instructions but are available from the corresponding author upon reasonable request.

## Supplementary Materials

The following supporting information can be downloaded at: https://www.mdpi.com/article/10.3390/pharmaceutics15092301/s1, Figure S1: Cytotoxicity study of MSCExo-Ce; Figure S2: Distribution of MSCExo and MSCExo-Ce in vivo.
