# Supplementary material for: Combining MSC Exosomes and Cerium Oxide Nanocrystals for Enhanced Dry Eye Syndrome Therapy
Source: Pharmaceutics. 2023 Sep 11;15(9):2301. doi: 10.3390/pharmaceutics15092301 (PMC10536361; doi:10.3390/pharmaceutics15092301)
Supplement: Supplementary file 1 [file pharmaceutics-15-02301-s001.zip › pharmaceutics-2545415-supplementary.pdf]

Supplementary information

Combining MSC Exosomes and Cerium Oxide Nanocrystals for Enhanced Dry Eye Syndrome Therapy

Ying Tian 1#, Yiquan Zhang 1#, Jiawei Zhao 1, Fuxiao Luan 1, Yingjie Wang 1, Fan Lai 2, Defang Ouyang 3, Yong Tao 1*

<u>Content</u>

Supplementary Figure S1. Cytotoxicity study of MSCExo-Ce
Supplementary Figure S2. Distribution of MSCExo and MSCExo-Ce in vivo

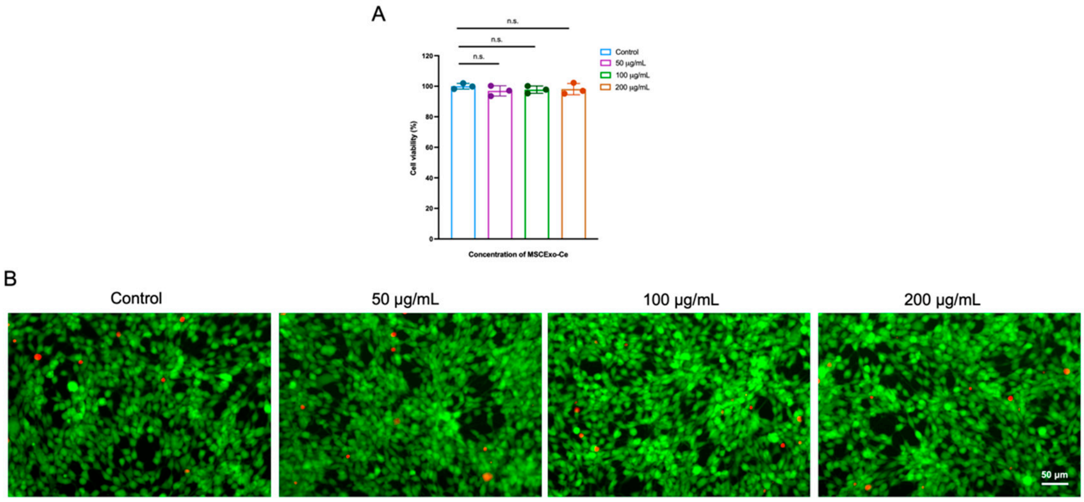

Figure S1. Cytotoxicity study of MSCExo-Ce

A. Viability of HCECs treated with different concentrations of MSCExo-Ce. Data are presented as the mean ± SD and are assessed via one-way ANOVA. (n.s. means not significant)

B. Live/dead analysis of HCECs treated with different concentrations of MSCExo-Ce. (green: live cells, red: dead cells). The scale bar is 50 μm.

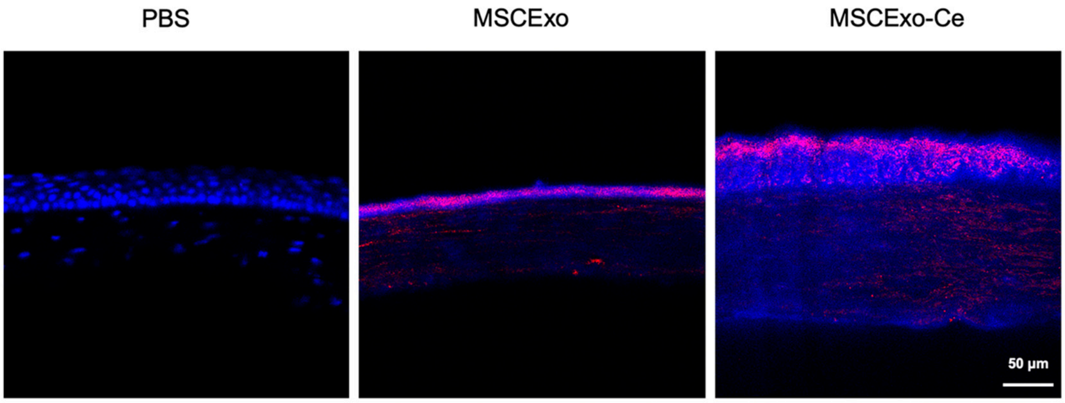

Figure S2. Distribution of MSCExo and MSCExo-Ce in vivo

Confocal laser scanning microscope (CLSM) images of distribution of MSCExo or MSCExo-Ce in the ocular surface of mice (red: Cy5-labelled MSCExo or MSCExo-Ce, blue: DAPI-labelled cell nucleus). The scale bar is 50 μm.
